# Isolated receptor binding domains of HTLV-1 and HTLV-2 envelopes bind Glut-1 on activated CD4+ and CD8+ T cells

**DOI:** 10.1186/1742-4690-4-31

**Published:** 2007-05-15

**Authors:** Sandrina Kinet, Louise Swainson, Madakasira Lavanya, Cedric Mongellaz, Amélie Montel-Hagen, Marco Craveiro, Nicolas Manel, Jean-Luc Battini, Marc Sitbon, Naomi Taylor

**Affiliations:** 1 Institut de Génétique Moléculaire de Montpellier (IGMM), 1919 Rte de Mende, F-34293 Montpellier Cedex 5, France; 2 CNRS, Montpellier, France; 3 Université Montpellier 2, IFR122, Montpellier, France; 4 Present address : Skirball Institute of Biomolecular Medicine, NYU School of Medicine, NY, NY 10016, USA

## Abstract

**Background:**

We previously identified the glucose transporter Glut-1, a member of the multimembrane-spanning facilitative nutrient transporter family, as a receptor for both HTLV-1 and HTLV-2. However, a recent report concluded that Glut-1 cannot serve as a receptor for HTLV-1 on CD4 T cells: This was based mainly on their inability to detect Glut-1 on this lymphocyte subset using the commercial antibody mAb1418. It was therefore of significant interest to thoroughly assess Glut-1 expression on CD4 and CD8 T cells, and its association with HTLV-1 and -2 envelope binding.

**Results:**

As previously reported, ectopic expression of Glut-1 but not Glut-3 resulted in significantly augmented binding of tagged proteins harboring the receptor binding domains of either HTLV-1 or HTLV-2 envelope glycoproteins (H1_RBD _or H2_RBD_). Using antibodies raised against the carboxy-terminal peptide of Glut-1, we found that Glut-1 expression was significantly increased in both CD4 and CD8 cells following TCR stimulation. Corresponding increases in the binding of H1_RBD _as well as H2_RBD_, not detected on quiescent T cells, were observed following TCR engagement. Furthermore, increased Glut-1 expression was accompanied by a massive augmentation in glucose uptake in TCR-stimulated CD4 and CD8 lymphocytes. Finally, we determined that the apparent contradictory results obtained by Takenouchi et al were due to their monitoring of Glut-1 with a mAb that does not bind cells expressing endogenous Glut-1, including human erythrocytes that harbor 300,000 copies per cell.

**Conclusion:**

Transfection of Glut-1 directly correlates with the capacities of HTLV-1 and HTLV-2 envelope-derived ligands to bind cells. Moreover, Glut-1 is induced by TCR engagement, resulting in massive increases in glucose uptake and binding of HTLV-1 and -2 envelopes to both CD4 and CD8 T lymphocytes. Therefore, Glut-1 is a primary binding receptor for HTLV-1 and HTLV-2 envelopes on activated CD4 as well as CD8 lymphocytes.

## Background

We identified the ubiquitous glucose transporter Glut-1 as a receptor for deltaretrovirus HTLV-1 and HTLV-2 envelopes (Env), mediating viral binding and entry [1]. We further identified Glut-1 extracellular loop 6 (ECL6) as the primary binding site for both HTLV-1 and 2 receptor binding domains (RBD) [2]. The identity of the HTLV Env receptor remained elusive for approximately two decades and the search was hindered by the fact that HTLV entry can take place in all established vertebrate cell lines and generally produces a rampant syncytial effect. This long search has been the source of numerous speculations as to the nature of the receptor, including the possibility that a dedicated cellular receptor may not be required for HTLV infection or that many different receptors can be used by HTLV [3]. The elucidation of the modular organization of the HTLV Env is based on that of a gammaretrovirus Env [4]: The identification and generation of tagged fusion proteins that comprise the RBD of the HTLV-1 and -2 Env, in the absence of the carboxy terminal domain [4,5], were essential to our finding that Glut-1 is a receptor for HTLV Env.

Biochemical studies assessing cell surface Glut-1 have been hampered by the lack of antibodies recognizing extracellular determinants of this transporter. This difficulty was in large part due to the high degree of homology between the Glut-1 extracellular domains of diverse mammalian species and indeed, studies aimed at generating antibodies to all domains of Glut-1 concluded that the extracellular loops are non-antigenic [6,7]. Notably, this high conservation of Glut-1 is likely responsible for the ability of HTLV-1 to infect all tested vertebrate cell lines. Recently though, a monoclonal antibody promoted as recognizing an extracellular domain of Glut-1, thereby allowing detection of surface Glut-1, has been made commercially available (RnD systems, mAb1418). Using this antibody, Takenouchi et al. did not detect binding on quiescent or activated CD4 T lymphocytes, a major reservoir of HTLV *in vivo*, leading these authors to question the role of Glut-1 as primary binding receptor for HTLV [8].

Numerous biochemical and cell biology experiments from our laboratory and others strongly support the role of Glut-1 as a receptor for both HTLV-1 and HTLV-2 [1,2,5,9-12]. It was therefore of significant interest to reassess Glut-1 expression on quiescent and activated CD4 as well as CD8 T cells as well as to analyze the relevance of the mAb1418 with regards to detection of Glut-1 expression.

## Results and discussion

### Binding of Glut-1 antibodies and H_RBD_-derived ligands to 293T cells transfected with Glut-1 and Glut-3 glucose transporters

**Figure 1:**
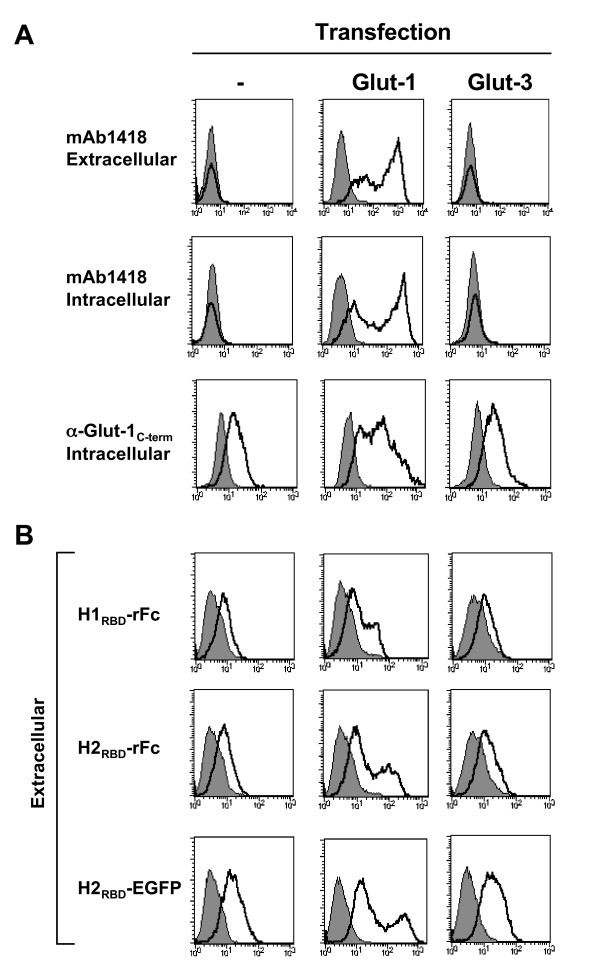
**Antibody and H_RBD _binding following transfection of the Glut-1 and Glut-3 glucose transporters**. (A) 293T cells were transfected with Glut-1 or Glut-3 expression vectors and assayed for binding to the mAb1418 and C-term anti-Glut-1 polyclonal Ab. The former stainings were performed on whole cells as well as permeabilized cells to determine cell surface and total binding, respectively. All stainings using the anti-Glut-1 pAb were performed on permeabilized cells as the recognized epitope is intracellular. Staining was performed at 4°C. Specific binding and background fluorescence due to the secondary conjugated Ab are indicated in solid line and filled histograms, respectively. (B) Control and transfected 293T cells were incubated with rFc-tagged H1_RBD _and H2_RBD _fusion proteins for 30 min at 37°C followed by incubation with a FITC-conjugated αrabbit-Fc antibody at 4°C. Direct binding to H2_RBD _was demonstrated by incubation of cells with an EGFP-tagged envelope (H2_RBD_-EGFP). Binding is shown in solid line histograms whereas control immunofluorescence is shown in filled histograms.

Takenouchi and colleagues used the commercially available antibody mAb1418 to conclude that Glut-1 was not expressed at the cell surface of CD4 T cells [8]. In order to assess the specificity of this antibody, we first determined its binding to cells transfected with either Glut-1 or Glut-3. Glut-3 is the closest isoform of Glut-1 and has similar glucose transport kinetics [13,14]. Flow cytometry analyses of Glut-1-transfected 293T cells stained with mAb1418 revealed a high level binding that was not detected following transfection with Glut-3 (Fig. 1A). However, no staining of endogenous Glut-1 was observed on these cells. This was concerning as the vast majority of transformed cell lines have been reported to express Glut-1. We therefore next assessed intracellular binding of mAb1418 following permeabilization but did not detect any significant changes as compared to the extracellular binding profile. This was also concerning as two populations of positive-binding cells were detected after Glut-1 transfection while no binding was detected on the initial population.

As mentioned above, even though antibodies directed against Glut-1 extracellular epitopes are problematic to obtain, polyclonal and monoclonal antibodies directed against the intracellular carboxy terminus tail (C-term) have been reported by several teams [6,15-18]. Indeed, using this type of intracellular polyclonal antibody directed against the C-term domain of Glut-1, we detected the expected baseline expression of endogenous Glut-1 in 293T cells. Moreover, an increase in Glut-1 staining was revealed following Glut-1 transfection (Fig. 1A). As 293T cells are known to express Glut-1 (see below), these data indicate that either the level of endogenous Glut-1 was below the level of detection necessary for mAb1418 staining or alternatively, mAb1418 does not recognize endogenous Glut-1 on these cells.

Notably, the ability of HTLV-1 and HTLV-2 derived RBDs to bind to parental and transfected 293T cells correlated with the data obtained using the C-term Glut-1 antibody. As we expected from previous studies, extracellular binding of rFc-fusion proteins encoding either H1_RBD _or H2_RBD _was significantly augmented in cells transfected with Glut-1 but not Glut-3 (Fig. 1B). Additionally, an H2_RBD_-EGFP fusion protein, that was concentrated to promote maximal binding, showed a similar profile (Fig. 1B).

These results contrast with those reported by Takenouchi et al., who did not detect increased binding to Glut-1-transfected COS-7 cells when the entire HTLV-1 SU was used as a ligand [8]. Significantly though, these latter binding studies were performed on ice, and in our hands, neither H1_RBD _nor H2_RBD _bind to Glut-1-expressing cells under those conditions (data not shown). Rather, in absence of fixation, binding of the HTLV RBDs requires incubation at physiological conditions, namely 37°C.

**Figure 2:**
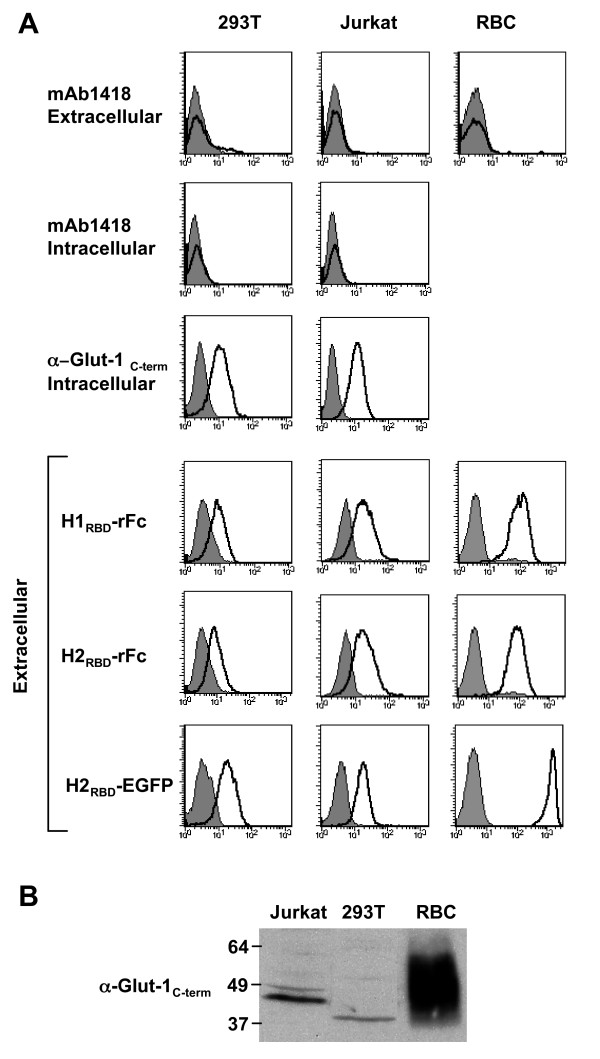
**Endogenous Glut-1 expression in diverse cell types is not reflected by mAb1418 reactivity but correlates with binding of the HTLV-1 and HTLV-2 Env RBDs**. (A) 293T, Jurkat and primary human erythrocytes were stained with mAb1418 and control binding with the secondary FITC-conjugated antibody is shown in all histograms (filled). Intracellular Glut-1 levels in permeabilized 293T and Jurkat cells were monitored with mAb1418 as well as the C-term polyclonal Glut-1 antibody. Expression of the HTLV-1 and HTLV-2 receptor was monitored by incubation of cells for 30 min at 37°C with the rFc-tagged H1_RBD _and H2_RBD _fusion proteins as well as the H2_RBD_-EGFP fusion protein. Binding is shown in solid line histograms whereas control immunofluorescence is shown in filled histograms. (B) Total Glut-1 protein levels in cell extracts from 293T, Jurkat and human erythrocytes were monitored by immunoblotting with an anti-C-term Glut-1 antibody.

To determine whether the absence of detectable intracellular or extracellular binding of mAb1418 to 293T cells was related to potentially low Glut-1 levels, we compared binding to a cell line expressing similar levels of this transporter, the Jurkat leukemic T cell line, and a cell type expressing significantly higher Glut-1 levels, human erythrocytes. The latter cells express the highest known levels of Glut-1 with 300,000 copies per cell; accounting for 10% of the total protein membrane mass [15,19]. In this context, it was therefore most remarkable that no detectable extracellular or intracellular staining was observed with mAb1418 on either Jurkat leukemia cells or human erythrocytes (Fig. 2A). In contrast, intracellular Glut-1 expression was detected in both 293T and Jurkat cells using the C-term Glut-1 antibody (erythrocytes cannot be analyzed by flow cytometry following permeabilization but were further assessed by immunoblotting as described below).

In contrast to a lack of extracellular or intracellular staining by mAb1418, both H1_RBD _and H2_RBD _interacted with extracellular Glut-1 resulting in significantly higher mean fluorescence intensity bindings to human erythrocytes as compared to either the 293T or Jurkat cell lines (Fig. 2A). Moreover, the level of H1_RBD _and H2_RBD _binding, but not that of mAb1418 staining, correlated directly with the total cellular levels of Glut-1 as detected by immunoblot (Fig. 2B). It should be noted that changes in the mobility of Glut-1 and the appearance of a smear in erythrocytes are well known and have been shown to be due in part to post-translational N-glycosylation modifications [15]. Indeed, treatment of cells with tunicamycin, a drug that inhibits N-linked glycosylation, or trimming of N-linked glycans with N-glycosidase F increases the electrophoretic mobility of Glut-1 [18].

These data lead to the question as to how the mAb1418 antibody was selected as being "specific" to Glut-1. According to the manufacturer's indications, this antibody was generated by immunization of mice with a murine cell line transfected with human Glut-1. The criterion for hybridoma selection was based on the ability to bind the transfected cell line and not the parental cell line. Thus, the capacity of this antibody to recognize endogenous Glut-1 was not specifically the basis for selection. The ensemble of the data presented here strongly indicates that mAb1418 does not detect endogenous Glut-1 but rather interacts with a cell surface protein that is associated with Glut-1 overexpression in transformed cell lines.

### TCR-induced expression of Glut-1 on both CD4+ and CD8+ T cells results in H1_RBD_/H2_RBD _binding and glucose uptake

**Figure 3:**
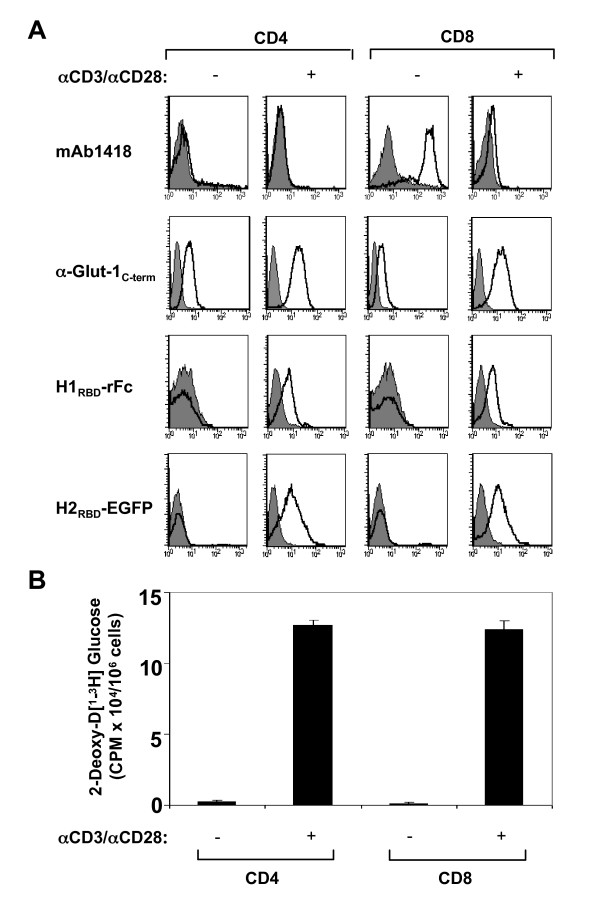
**TCR stimulation results in Glut-1 expression and concomitant glucose uptake in CD4 and CD8 lymphocytes: Induction of H1_RBD _and H2_RBD _binding**. CD4+ and CD8+ T lymphocytes were isolated by negative selection and stimulated via the TCR using αCD3/αCD28 mAbs. (A) Non-activated and TCR-activated T cells were used for binding assays with mAb1418 followed by incubation with a FITC-conjugated αmouse IgG. Intracellular Glut-1 levels were monitored in permeabilized cells using the C-term Glut-1 polyclonal antibody followed by incubation with a FITC-conjugated sheep αrabbit IgG antibody. Filled histograms depict binding in the presence of the secondary FITC-conjugated antibody alone. Expression of the HTLV-1 Env receptor was detected by a 30 min incubation of the non-activated and TCR-activated cells with rabbit rFc-tagged H1_RBD _fusion protein at 37°C and binding was revealed by a 20 min incubation at 4°C with a FITC-conjugated sheep αrabbit IgG antibody. Binding to the H2_RBD _domain fused directly to EGFP (H2_RBD_-EGFP) was detected following a 30 min incubation at 37°C. (B) Glucose uptake was assayed by incubating non-activated and TCR-activated CD4 and CD8 T cells (1 × 10^6^) with 2-deoxy-D [1-^3^H]glucose (2 μCi) for 45 min at 37°C. Uptake for each cell population is expressed as mean counts per minute (CPM) for triplicate samples, error bars indicate SD.

The premise of Takenouchi et al. that Glut-1 cannot serve as a primary binding receptor for HTLV on CD4 T lymphocytes was based largely on their supposition that the Glut-1 transporter is not expressed on this lymphocyte subset [8]. Indeed, using mAb1418, the antibody which served as the basis for their conclusions, we also did not detect any staining of either quiescent or TCR-activated CD4 T cells. We also observed significant mAb1418 staining of quiescent CD8 T cells (Fig. 3A). In the experiments reported here, staining of CD8 lymphocytes decreased significantly following TCR engagement (Fig. 3A) whereas Takenouchi et al. observed stable mAb1418 staining after their ex vivo stimulation protocol. In the previous studies, activations were performed using phytohemagglutinin (PHA) alone, and it is likely that at 48 h post-stimulation, the time point at which analyses were performed, the vast majority of lymphocytes were not in a fully active state. In a representative experiment where we compared PHA activation with the anti-CD3/CD28 antibody stimulation used here, the percentages of cells that had entered into cycle (G1b/S/G2/M) were 16% and 81%, respectively (data not shown). Thus, mAb1418 staining is significantly decreased following optimal TCR stimulation of CD8 T lymphocytes.

These data are in complete contradiction with the long-standing notions that; 1) quiescent T cells demonstrate very low glucose transport and 2) the energy demands of an activated T cell are met by an increase in glucose transport as well as metabolism [20-23]. The main functional glucose transporter isoform on CD4 as well as CD8 T lymphocytes has been reported to be Glut-1. In fact, a large body of work by several laboratories has established that Glut-1 is expressed at low levels on quiescent T cells but is induced upon T cell receptor activation as well as cytokine stimulation [9,16,24-29]. Moreover, in the absence of stimulation or transformation, surface Glut-1 is not detected on T cells of naïve, memory or effector phenotypes [9,16].

To further assess Glut-1 expression on quiescent and activated T cell subsets, we used the intracellular C-term anti-Glut-1 polyclonal antibody raised against a peptide encoding the 13 C-terminal amino acids. As expected from the literature cited above, Glut-1 expression was significantly increased in both CD4 and CD8 T cells following TCR stimulation (Fig. 3A), a time at which these cells had become blast-like and expressed activation markers such as CD25 and CD69 (data not shown). In parallel, binding of H1_RBD _as well as H2_RBD_, not detected on quiescent T cells, was clearly augmented following TCR stimulation. These data are in contradiction with that of Jones and colleagues who do not detect binding of the HTLV-2 SU to activated CD4 T cells [30], but several technical differences in the receptor binding assays are likely to explain this discrepancy. Indeed, we use isolated RBDs, in the absence of the adjacent Env SU domains that may alter primary receptor binding. Also, our RBDs are produced as soluble secreted proteins, whereas Jones et al. use entire SU immunoadhesins prepared from sonicated whole cell extracts which are likely to contain heterogeneous post-translationally modified proteins. Furthermore, we performed RBD binding assays at 37°C as neither binding of H1_RBD _nor H2_RBD _is detected at 4°C (data not shown). Notably, this is also the case for many RBDs from gammaretroviruses, including amphotropic, xenotropic, and FeLV-C (our unpublished observations). Using the SU immunoadhesins, Jones et al. perform their HTLV envelope binding assays at 4°C on fixed cells.

Staining with the anti-Glut-1 polyclonal antibody revealed low binding to permeabilized quiescent CD4 and CD8 T cells while no detectable surface staining was observed on either quiescent cell types when using H_RBD _ligands. This difference may be due to cell permeabilization prior to antibody staining, resulting in the recognition of a small intracellular pool of Glut-1 that is not present at the cell surface. In support of the latter hypothesis, Baldwin and colleagues have elegantly shown that cytokines and other growth signals induce the translocation of cytoplasmic Glut-1 to the cell surface in several different cell models [31-33]. In this context, it is interesting to note that stable expression of GFP-tagged Glut-1 results in its accumulation in intravesicular pools, whereas Glut-3 appears almost exclusively expressed at the cell surface (data not shown).

The expected primary physiological consequence of cell surface Glut-1 expression is glucose transport. It was therefore of interest to assess glucose uptake on quiescent and activated CD4+ and CD8+ lymphocyte subsets. Glucose uptake, as measured by the ability of cells to uptake nonhydrolyzable 2-deoxy-D [1-^3^H]glucose, was at the limits of detection in both quiescent CD4 and CD8 T cells and increased by >20-fold in both subsets following stimulation (Fig. 3B). Notably, the increase in glucose uptake was equivalent in the CD4 and CD8 populations, demonstrating yet again that only activated lymphocytes express high surface levels of a glucose transporter. These results are in total agreement with other studies assessing glucose transporter expression in T lymphocytes [10,16,24,25]. Although it is not clear what cell surface protein is recognized by mAb1418 on quiescent CD8 T cells, these results indicate that the cognate antigen of mAb1418 is not a member of the glucose transporter family.

**Figure 4:**
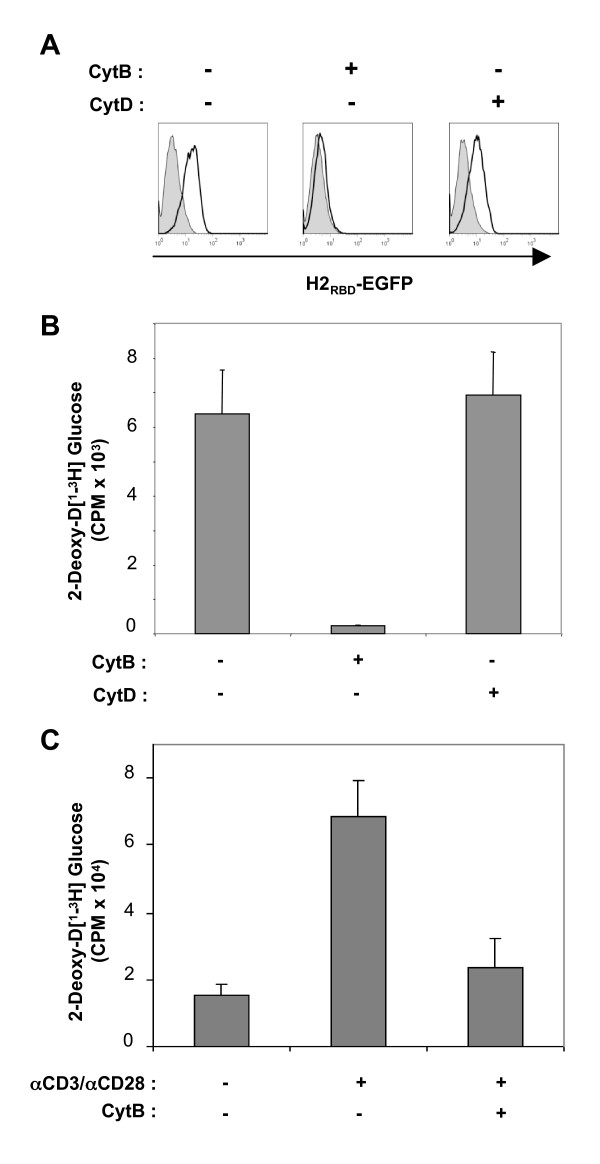
**Glucose uptake in CD4+ T cells is abrogated by the cytochalasin B Glut-1 inhibitor**. (A) H2_RBD_-EGFP binding to Jurkat T cells was assessed following incubation in the absence or presence of the Glut-1 inhibitor cytochalasin B (CytB,100 μM) or the related cytochalasin D (CytD, 100 μM) molecule for 30 min. Filled and solid line histograms depict control and H2_RBD_-EGFP binding, respectively. (B) Glucose uptake was assayed by incubation of control, cytochalasin B-, and cytochalasin D-treated Jurkat cells in the presence of 2-deoxy-D [1-^3^H]glucose (5 μM) for 10 min at room temperature. (C) Glucose uptake in quiescent CD4 T cells, CD3/CD28-activated CD4 T cells and cytochalasin B-treated CD3/CD28-activated CD4 T cells was assessed as described in the legend of figure 3. Uptake for each condition is expressed as mean counts per minute (CPM) for triplicate samples, error bars indicate SD.

To further assess whether surface Glut-1 on activated CD4 T cells and a transformed CD4+ T cell line, Jurkat, was responsible for glucose uptake in these cells, lymphocytes were first treated with cytochalasin B (CytB). This molecule inhibits Glut-1 function by directly binding to its sugar export site [34,35]. In the presence of CytB, H2_RBD_-EGFP binding was significantly decreased, strongly suggesting that CytB binding to Glut-1 directly inhibits binding of the HTLV envelope. This effect was specifically due to the action of CytB on Glut-1 and not to an indirect cytochalasin effect on microfilaments: Treatment of Jurkat cells with the related cytochalasin D (CytD) molecule, which is not a Glut-1 ligand, did not alter H2_RBD_-EGFP binding (Fig. 4A). Moreover, CytB, but not CytD, inhibited glucose uptake by CD4+ Jurkat cells as well as primary activated CD4 T cells by greater than 90% (Figs. 4B and 4C). Altogether, the data reported here show that surface Glut-1 as well as subsequent transporter function is upregulated on both CD4 and CD8 T cells that have been stimulated via the TCR.

## Conclusion

Induction of surface Glut-1, both upon exogenous transfection and stimulation of the endogenous protein, directly correlates with the capacities of HTLV-1 and HTLV-2 envelope-derived ligands to bind cells. We have previously reported a physical interaction between HTLV-1 and HTLV-2 RBDs and Glut-1 and mapped the primary binding site to ECL6 [2]. Moreover, down-modulation of endogenous Glut-1 by siRNAs resulted in a significant reduction in HTLV-1 and HTLV-2 Env binding as well as Env-mediated infection [1]. In theses systems, as well as in recently published studies by others, Glut-1 was found to serve as cell surface receptor for both HTLV-1 and -2 infection [1,2,10-12].

Retroviral entry has been extensively shown to depend on several steps. These include initial receptor binding ensured by RBD-harboring amino terminal domains of the SU, followed by subsequent conformational changes and Env-receptor complex remodeling that leads to the unmasking of the TM fusion peptide and membrane fusion, either at the cell surface or after endocytosis and acidification of endocytic vesicles. Using Glut-1 and Glut-3 chimeric molecules, we have previously shown that while the Glut-1 ECL6 was sufficient to confer HTLV Env binding to Glut-3, ECL1 and 5 were required for HTLV-Env-mediated infection [2]. Therefore, successful post-binding events involving HTLV Env regions located outside of H_RBD _in conjunction with Glut-1 ECL1 and 5 are likely to depend on other co-factors. In this regard, it is interesting to note that neuropilin-1 and HSC70 have been shown to interact with the SU region located immediately downstream of H_RBD _[12,36]. Heparan sulfates, which modulate the attachment of numerous viruses [3,37], are also likely to influence the surface environment conditioning HTLV entry following Env-Glut-1 interactions [38].

Therefore, it will be essential to more precisely define the cell surface environment in which Glut-1 is expressed in distinct cell types in order to determine the parameters that condition HTLV envelope binding and subsequent infection. Identification of cellular factors that modulate the localization of Glut-1 as well as its cell surface membrane microenvironment are likely to shed new insights into our global understanding of HTLV *in vivo *tropism and associated physiopathologies.

## Methods

### Cell transfections

293T cells were transfected with the Glut-1 and Glut-3 expression vectors using the calcium phosphate method. Human *Glut-1 *and *Glut-3 *cDNAs were amplified by PCR from the pLib HeLa cDNA library (Clontech), and inserted into pCHIX, a modified version of the pCSI vector that includes a C-terminal factor Xa cleavage site, and the hemagglutinin (HA) and histidine tags [1]. After an overnight transfection, cells were washed in phosphate-buffered saline (PBS), fresh medium was added and cells were cultured for an additional 24 h.

### T cell isolation and culture

To avoid artifactual stimulation, CD4+ and CD8+ T cells were each purified by negative selection using tetrameric complexes in which one antibody recognizes a surface antigen on B cells, monocytes, NK cells or CD8+ vs CD4+ cells, respectively, and the other recognizes glycophorin A on the surface of red blood cells (RosetteSep, StemSep Technologies, Vancouver, Canada). The non-purified cells were then pelleted upon ficoll-hypaque separation. The purity of the selected cells was monitored after each isolation on a FACSCalibur (BD Pharmingen, San Jose, CA, USA) following staining with FITC-conjugated αCD3 and phycoerythrin-conjugated αCD4 mAbs or -αCD8 mAbs (Beckman Coulter, Marseille, France). The purity of the selected cells was > 90%.

Lymphocytes were cultured in RPMI 1640 medium supplemented with 10% fetal calf serum (FCS, BioWest, France), penicillin and streptomycin. Cells were stimulated with immobilized αCD3 (UCHT-1) and αCD28mAbs (1 μg/ml) for two to four days.

### Generation of HTLV-1 and HTLV-2 RBD fusion proteins

The H1_215_SU and H2_178_SU subdomains, corresponding to the HTLV-1 and -2 SU amino terminus were generated by PCR and subcloned into the pCSI expression vector as fusion proteins harboring a carboxy terminal rFc tag [5], and are herein referred to as H1_RBD _and H2_RBD_, respectively. Additionally, the amino terminal 178 amino acids of the HTLV-2 RBD was fused to the EGFP coding sequence (from pEGFP-N3, Clontech) lacking the ATG initiation codon by PCR amplification in the pCSI expression vector and is herein referred to as H2_RBD_-EGFP. 293T cells were transfected with the indicated vector using a calcium-phosphate-Hepes buffered saline (HBS) transfection protocol. Transfection medium was replaced with fresh culture medium twenty hours post-transfection. Forty-eight hours post-transfection cell culture medium (supernatant) was recovered and filtered through a 0.45 μm pore-size membrane to remove cell debris. H2_RBD_-EGFP supernatants were concentrated 50–100-fold by filtration at 4°C on an iCON Concentrator 20 K (Pierce, Rockford, IL, USA). Supernatants were stored at -20°C until further use.

### Flow cytometry

Staining with the mAb1418 (RnD Systems) was performed by incubating 1 × 10^5 ^cells for 20 min on ice at 1:25 to 1:100 antibody dilutions followed by staining with a FITC-conjugated goat anti mouse IgG (Sigma) at a 1:100 dilution. Background fluorescence was measured following staining with the secondary antibody alone. Staining with the anti-carboxy terminal Glut-1 polyclonal antibody (generously provided by A. Carruthers) was performed following intracellular staining by fixation (Cytofix Cytoperm solution, BD Pharmingen) and permeabilization (PhosFlow Perm III, BD Pharmingen). The secondary antibody was a FITC-conjugated goat anti-rabbit IgG (Sigma). For H_RBD _stainings, 1–5 × 10^5 ^cells were incubated at 37°C for 30 min with 100–500 μls of either undiluted H_RBD_-rFc supernatants or a 1:25–1:50 dilution of the concentrated H2_RBD_-EGFP ligand. Cells were then washed with PBA (2% FCS and 0.01% sodium azide), and for H1_RBD _and H2_RBD _stainings, cells were incubated with a FITC-conjugated sheep anti-rabbit Fc antibody (1:500 dilution; Sigma) for 20 min on ice. Cells were analysed on a FACSCalibur flow cytometer (Becton Dickinson). Data analyses were performed using CellQuest Pro (Becton Dickinson) or FlowJo (TreeStar) software.

### Immunoblotting

Non-boiled lysates from 293T, Jurkat, and human erythrocytes were electrophoresed in SDS-10% acrylamide gels, transferred and probed with the anticarboxy terminal Glut-1 antibody (1:10,000) followed by a peroxidase-conjugated anti-rabbit immunoglobulin antiserum. Proteins were visualized using the ECLplus kit (Amersham).

### Glucose uptake

5 × 10^5 ^CD4+ or CD8+ T cells were incubated at 37°C in serum-free RPMI for 30 min, then washed and incubated for an additional 30 min in serum/glucose-free RPMI. Uptake was initiated by adding labeled 2-deoxy-D [1-^3^H]glucose (Amersham Biosciences; 2 μCi/ml) to an unlabelled deoxy-glucose concentration of 0.1 mM and incubating cells for 45 min at 37°C. Alternatively, glucose uptake was performed in a 50 μl volume in the presence of 5 μM 2-deoxy-D [1-^3^H]glucose (2 μCi) for 10 min at RT. Cells were then washed in cold serum/glucose-free RPMI, and solubilized in 0.1% SDS. Radioactivity was measured by liquid scintillation and statistical analyses were performed using Student's t test.

## Competing interests

The H2_RBD_-EGFP is commercially available without any private interest to the authors of the manuscript.

## Authors' contributions

SK and LS participated in the design of the study, performed significant numbers of the experiments and helped with the draft of the manuscript; LM, CM, AMH, and MC participated in the design and validation of antibodies and receptor binding domain constructs as well as binding studies; NM helped to conceive the studies, participated in their design and performed the initial comparisons of RBDs; NT is responsible for the overall study and drafted the manuscript together with MS and JLB. All authors read and approved the final manuscript.
